# Minimally invasive delivery of ethanol for the treatment of urinary bladder fistulas

**DOI:** 10.1259/bjrcr.20210239

**Published:** 2022-04-25

**Authors:** Andrew Nanapragasam, Syed Umair Mahmood, Sebastian Mafeld, Philip Haslam

**Affiliations:** 1 Department of Medical Imaging, University of Toronto, Toronto, Ontario, Canada; 2 St. Joseph’s Hamilton Healthcare, Hamilton, Ontario, Canada; 3 Department of Medical Imaging, University Health Network, Toronto General Hospital, Toronto, Ontario, Canada; 4 The Newcastle Hospitals NHS Foundation Trust, Newcastle-upon-Tyne, United Kingdom

## Abstract

Urinary bladder fistula formation is a complication of significant morbidity and mortality following pelvic surgery or radiotherapy. Surgical treatment is the definitive management, but it may be contraindicated in patients with significant comorbidities. The alternative approach is to divert urine away from the fistula with stents and catheters, and allow time for healing. The case illustrated herein describes the use of alcohol to accelerate the fibrotic healing of a urinary bladder fistula, based on the premise that sclerosing agents have been effective in the treatment of pancreatic fistulas and renal cysts.

A Foley catheter is inserted through the external fistula orifice and passed along the fistula tract into the urinary bladder. The Foley catheter balloon is inflated and pulled back to occlude the fistula. Following this, a vascular sheath is placed alongside the catheter and ethanol is injected into the tract. The alcohol is left to dwell in the fistula for a few minutes, after which time the catheter and sheath are removed. The sclerosant effect of the ethanol aims to induce fibrosis, and therefore occlusion, of the fistula.

## Introduction

Urinary bladder fistula formation can occur as a complication of pelvic radiotherapy or surgery, or as a consequence of a traumatic injury.^1,2^ The local anatomy around the urinary bladder has many structures to which a fistulous tract can communicate, including the bowel, the uterus, the vagina, and the skin.^3^ Such fistulous connections are a source of significant distress and morbidity for those patients who develop this condition.

Determining the optimal treatment for these fistulas is a challenge as definitive treatment often requires a surgical intervention, which can be precluded by the patient’s comorbidities, or complicated by perioperative morbidity and/or failure to achieve a long-term cure. A non-surgical approach to urinary fistula management is sometimes considered, aiming to promote urinary excretion via the normal anatomical pathway, while limiting the time for which the irritative urine is in contact with the fistulous tract. In order to achieve this, bilateral nephrostomies, bilateral double “J” stents, and a long-term urinary catheter may be required. This treatment strategy entails a protracted recovery period as the treatment relies upon the body’s capacity to heal. The non-surgical “urinary diversion” approach is preferred for those patients who are not deemed to have the necessary physiological reserve or desire to undergo a corrective surgery. This approach is also preferred in those patients who have undergone multiple surgeries in the past, as a repeat surgical intervention is not only technically challenging, but also likely to increase inflammation and may predispose to more fistulas. However, even those patients deemed suitable for surgical intervention are not guaranteed a long-term cure.^4–6^

Given the challenging nature of this condition and the imperfect solutions, an alternative minimally invasive, radiological approach has been considered in those patients who are not candidates for operative treatment. Interventional radiology allows delivery of targeted therapy, aimed to accelerate the fistula tract closure, without incurring the complications of open surgery.

### Existing evidence for the minimally invasive treatment of urinary bladder fistulas

There are few reports of the percutaneous treatment of urinary bladder fistulas, as such, the evidence base is limited. Mechanical closure of ureteric fistulas with stents, tissue adhesives, plugs, and balloons have been attempted.^3,7^ While the placement of a physical device to achieve closure provides an immediate solution, its long-term success relies upon its position being maintained, which is not guaranteed.^1^ Furthermore, the aforementioned techniques are specifically tailored to the treatment of ureteric fistulas, and cannot be readily translated to the treatment of urinary bladder fistulas. This is because the successful mechanical closure of the ureteric fistula is predicated on the presence of the adjacent tubular ureter, to which the various closure devices are secured.

An alternative approach to the percutaneous mechanical closure of ureteric fistulas can be achieved through electrofulguration.^3,7^ Electrofulguration uses an alternating electric current to generate heat and cauterise the ureteral tissue, thereby sealing off the fistula. The principles of this technique for ureteric fistula treatment could theoretically be applied to bladder treatment. Indeed, there is some precedence in the literature for cauterisation of a urinary bladder fistula.^8^ There are some difficulties with this approach, for instance, failure to achieve long-term closure of the urinary bladder fistula is a recognised complication. Furthermore, this technique is not always technically feasible. If the fistulous tract is convoluted then achieving adequate contact with the electrocautery probe to the entire undulating contour of the fistula may prove difficult. However, when successful, the induction of tissue necrosis and the subsequent fibrosis with electrocautery can potentially achieve occlusion of the aberrant connection. Seeking to emulate the mechanistic benefits of electrocautery, but with fewer technical contraindications, an alternative means of closing bladder fistulas with alcohol (99% ethanol) ablation was considered.

Alcohol ablative solutions are a recognised treatment option for pancreatic fistulas.^9,10^ The alcohol is thought to induce necrosis by cellular dehydration, which in turn precipitates fibroblast proliferation, thereby sealing the fistulous defect.^11,12^ Alcohol has been used for the sclerotherapy of various other structures, including renal/hepatic cysts, aneurysmal bone cysts, arteriovenous malformations, and lymphangiomas.^12^ The use of alcohol in the treatment of renal cysts is perhaps the most well-established and accepted example of the use of alcohol as a therapeutic inducer of the body’s sclerosing mechanism. Side effects noted with alcohol sclerotherapy include localised pain, hypersensitivity, and tissue necrosis in adjacent structures. Systemic complications are rare but include anaphylactic reactions, and, if alcohol enters the blood vessels, thromboembolism.^12^

With this evidence in support of alcohol, consideration can be given to its use in the treatment of urinary fistulas, specifically in comparison to electrocautery. A disadvantage of electrocautery is the difficulty in achieving close contact between the probe and the irregular contour of the fistula, which would increase the risk of incomplete fistula closure. As the alcohol would be delivered in a liquid formulation it ought to fill the fistulous tract and achieve good contact with the entirety of fistula’s wall. The use of alcohol to treat urinary bladder fistulas is likely to share a similar set of side effects as those incurred with alcohol sclerotherapy. In order to limit the occurrence of such complications, it is advisable to target the delivery of the alcohol to only the tissue that is to be treated. Guidewires and inflatable balloons are a possible means of isolating the fistula tract. Inflating a balloon creates a seal compartmentalising the aberrant fistulous connection from the adjoining normal bladder. The alcohol can then be delivered in a targeted manner to the fistulous segment. Cautious instillation of alcohol avoids overfilling the cavity and further reduces the incidence of complications. It is inevitable that some of the alcohol will overflow from the fistula when the percutaneous access apparatus is removed. The consequences of this spillage into the urinary bladder can be limited by keeping some fluid within the bladder by temporarily clamping the catheter or instilling some saline. Even a small amount of fluid within the bladder will immediately dilute the alcohol that might leak from the fistula tract, thereby curtailing its necrotic properties, and allowing it to be safely excreted from the body via a urinary catheter.

This interventional approach is not considered an immediate cure, as it merely aims to accelerate the fistula tract closure for those patients who are not surgical candidates. As with other patients who receive a non-surgical treatment of their fistula, the fistula can persist, but the theoretical aim is to reduce the time required for healing. Repeat attempts at alcohol ablations many also help with the treatment, for those patients who suffer a recurrence of their fistula. A persistent or recurrent fistula is considered more likely if the tract is longstanding or is particularly wide. The need for multiple interventions is not unique to bladder fistula treatment, as ethanol sclerotherapy for the treatment of renal cysts often requires multiple interventions for long-term control.^13^

## Method and technique

**Figure 1.**
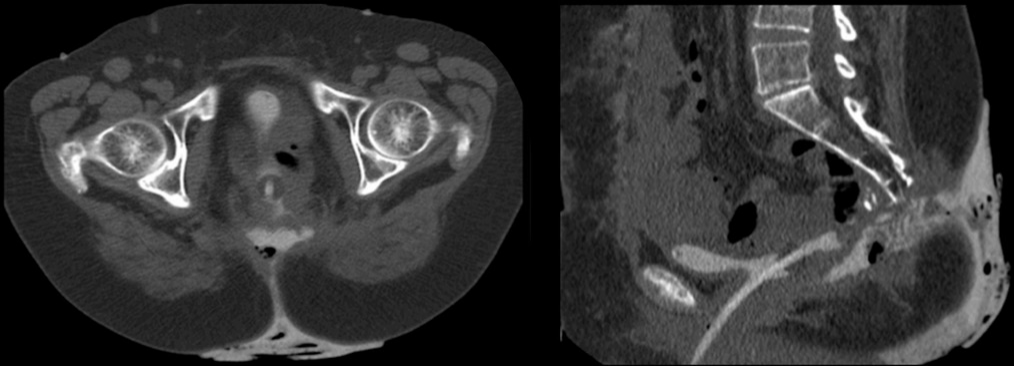
Axial (**A**) and sagittal (**B**) CT pelvis following contrast injection via a Foley catheter shows the posteriorly sited urinary bladder wall defect.

A CT examination is an essential step in the assessment of urinary bladder fistulas, providing important anatomic information on the fistula and its communications. A CT examination following contrast injection through the urinary catheter (Figure 1A, B) illustrates a posteriorly sited urinary bladder fistula that communicates with the natal cleft skin. In addition, an intravenous contrast-enhanced CT is advised for the exclusion of malignancy in the fistula. The illustrated vesicocutaneous fistula developed in a 52-year-old female following a colectomy for rectal adenocarcinoma and initial attempts at surgical correction were not successful. Conservative management with a Foley catheter and bilateral nephrostomies for 6 months had failed.

Although CT examination is an important diagnostic tool, the morphology of the defect often needs confirmation with fluoroscopy prior to ethanol ablation, as this dynamic study is better suited to accurately delineating the contour, trajectory, and calibre of the defect. Diluted contrast is slowly infiltrated into the bladder via a urethral Foley catheter with sequential images being taken to delineate the fistulous tract. As fistulous tracts may be multifaceted, thorough identification of all aberrant communications is essential for the successful closure of the fistula.

The procedure is performed under general anaesthesia with the patient lying prone. A Foley catheter is passed through the external cutaneous fistula orifice and then into the urinary bladder. At this point, the urinary bladder’s fistulous orifice is obstructed by inflating the catheter balloon and pulling back gently on the Foley catheter until the internal fistula orifice is sealed. Depending on the tortuosity of the fistula, a Foley catheter may or may not be able to directly traverse the full length of the aberrant communication. In instances where the Foley catheter is unlikely to be successful, a guidewire can be used to navigate the fistula tract first, with the Foley catheter subsequently being inserted over the wire. Alternatively, an angioplasty or compliant vascular occlusion balloon may be used.

**Figure 2.**
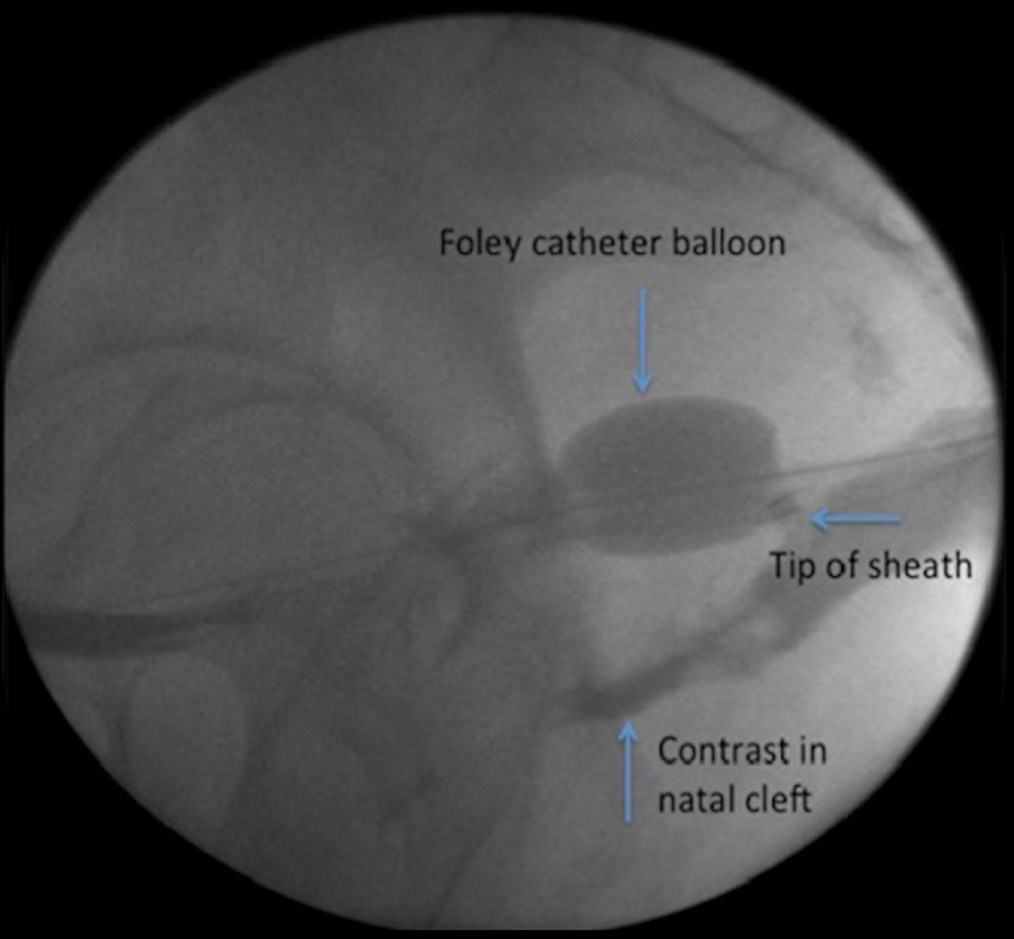
Fluoroscopic screen grab showing a small Foley catheter passed over a guidewire into the urinary bladder. The balloon was inflated and pulled back to occlude the fistula tract. Ethanol is then injected using a 5-F vascular sheath just superficial to the Foley catheter balloon.

Having anatomically isolated the fistula from the urinary bladder, a separate 5 French catheter or sheath is inserted through the external fistula orifice along the fistula tract until it reaches the Foley catheter balloon (Figure 2). Ethanol is then instilled into the fistula. By virtue of gravity and the occluding balloon, the ethanol is contained within the defect, and slowly fills the tract. The aim is to administer sufficient ethanol solution to fill the defect, but not so much that the ethanol spills over on to the normal tissue adjacent to the external orifice. The ethanol is left in contact with the fistula for a period of 5 min. This time may be sufficient to achieve fibrosis, however, an extended duration may be more efficacious especially for longer, more complex tracts. Practitioners should use their judgement when evaluating the risks and benefits of prolonged instillation times. In our experience, too short an instillation leads to the need for repeat interventions, but this has to be balanced with the risk of leakage of ethanol with longer instillation times. After this time has concluded, the ethanol is aspirated from the fistula as completely as possible.

Regardless of how well apposed the Foley catheter balloon is to the internal fistula orifice, some of the ethanol will inevitably seep past the balloon and enter the urinary bladder. Keeping the urinary bladder slightly distended with saline creates a reservoir of fluid which will dilute any ethanol that escapes from the fistulous tract, thereby minimising the adverse effect of the ethanol on the normal bladder tissue.

**Figure 3.**
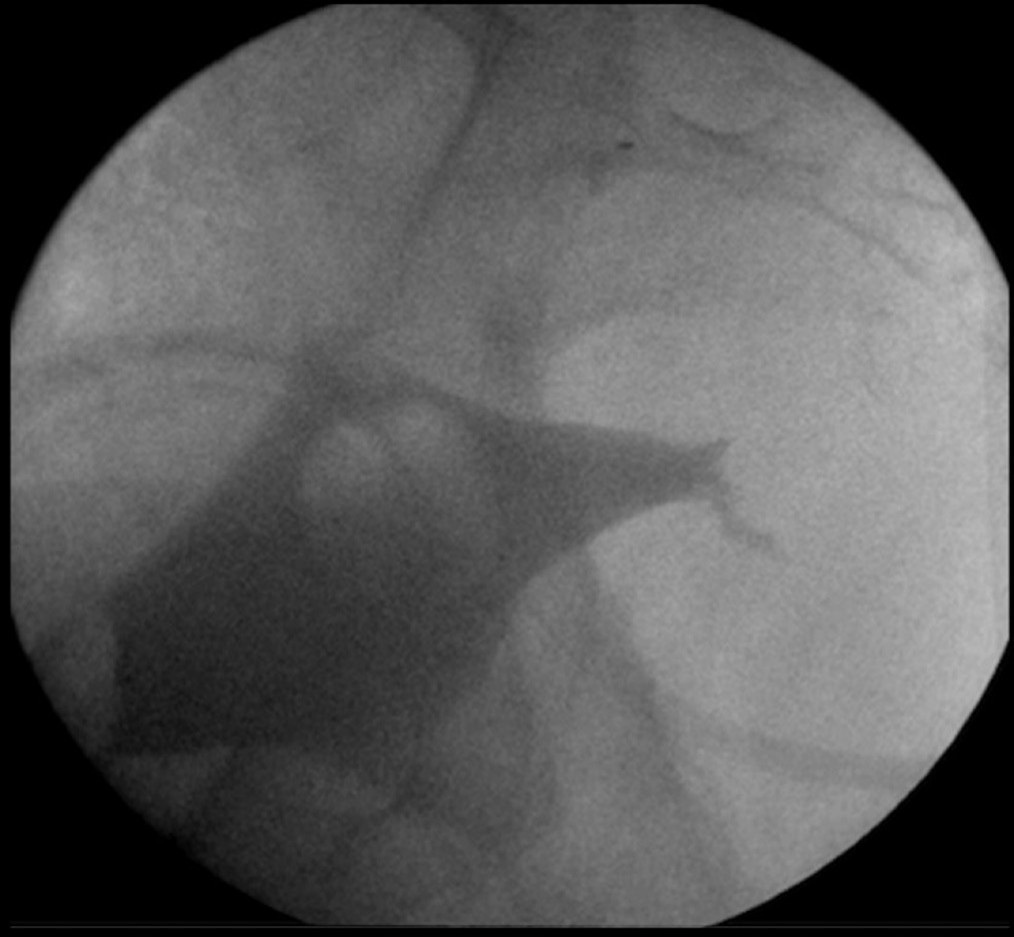
Fluoroscopic screen grab from a cystogram performed 4 weeks after the fistula treatment shows only a small, non-communicating remnant of the previously seen vesicocutaneous fistula.

**Figure 4.**
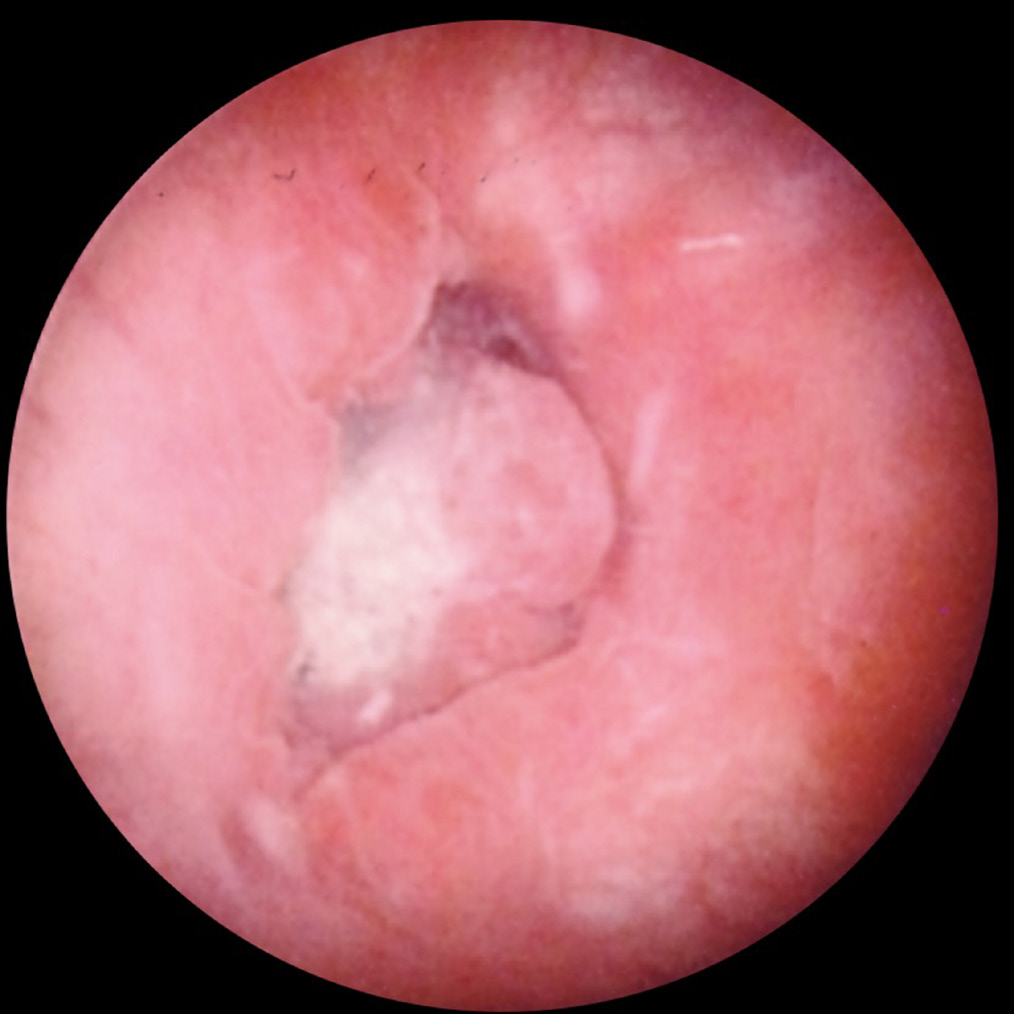
Cystoscopic view from within the urinary bladder showing the occluded fistula tract 4 weeks after treatment with ethanol.

There are no specific post-treatment instructions given to the patient, aside from the usual recommendations to keep the region clean and dry. The patient is closely followed up in the outpatient setting at intervals of 2–4 weeks. A follow-up cystogram (Figure 3) or endoscopic examination (Figure 4) may be performed in order to confirm the successful treatment of the urinary bladder fistula.

The case described in this report, is one of three patients who have benefitted from this minimally invasive technique. All three of these patients had previously undergone extensive pelvic surgery for malignancy resection which resulted in the formation of urinary bladder fistulas. Following the failure of conventional, conservative treatment strategies, an interventional approach was considered. Two of these patients had fistulas communicating with the skin, while the third patient’s fistula communicated with the lower rectum. The vesicorectal fistula was accessed via the low rectal orifice and treated in a similar manner to the technique described above. No procedural complications have been observed following the interventions in these three patients. With careful use of the described technique, in particular the filling of the bladder with saline, the potential for adverse effects with ethanol use can be reduced. As our knowledge of this technique is still in its infancy, a cautious approach is advised with regard to the selection of patients. At this time, patients considered for this procedure are limited to those with urinary bladder fistulas who have not responded to conventional treatment strategies. Further work in this area is advised to ensure reproducibility of this technique, and to assess whether with other fistulous connections may also benefit.

## Conclusions

The treatment of urinary bladder fistulas is notoriously challenging. Definitive surgical treatment of these patients is sometimes precluded by the operative risk, while conservative management strategies are confounded by their protracted healing times. This article demonstrates the potential of minimally invasive, image-guided alcohol injection as a means of accelerating the fibrotic occlusion of the aberrant tract. Our understanding of the benefit of this practice is still limited, and further investigation is advised before its widespread use.

## Learning points

- Urinary bladder fistulas can occur after pelvic surgery or radiotherapy, and they are often refractory to treatment with conventional surgical and conservative measures.
- The minimally invasive, targeted delivery of alcohol to ablate urinary tract fistulas may have value as treatment of fistulas that persist despite conventional therapy.
- Careful selection of patients is required as our understanding of this treatment’s efficacy is in its early stages
